# Whole Genome Sequencing of Extended-Spectrum Beta-Lactamase (ESBL)-Producing *Escherichia coli* Isolated From a Wastewater Treatment Plant in China

**DOI:** 10.3389/fmicb.2019.01797

**Published:** 2019-08-02

**Authors:** Xiawei Jiang, Xinjie Cui, Hao Xu, Wenhong Liu, Fangfang Tao, Tiejuan Shao, Xiaoping Pan, Beiwen Zheng

**Affiliations:** ^1^College of Basic Medical Sciences, Zhejiang Chinese Medical University, Hangzhou, China; ^2^Collaborative Innovation Center for Diagnosis and Treatment of Infectious Diseases, State Key Laboratory for Diagnosis and Treatment of Infectious Diseases, The First Affiliated Hospital, College of Medicine, Zhejiang University, Hangzhou, China

**Keywords:** waste water treatment plants, ESBL-producing *Escherichia coli*, antimicrobial resistance genes, metagenomics, *bla*_*CTX–M*_

## Abstract

**Background and Objectives:**

Wastewater treatment plants (WWTPs) are one of the major reservoirs for antimicrobial resistant bacteria (ARB) and antimicrobial resistance genes (ARGs) in the environment. Thus, the investigation on ARB and ARGs from WWTPs has attracted increasing attention in recent years. In order to uncover the resistome in a WWTP treating effluents from a pharmaceutical industry in China, the extended-spectrum beta-lactamase (ESBL)-producing *Escherichia coli* strains were isolated and their whole genome sequences were obtained and analyzed. Moreover, metagenomic sequencing was applied to give a comprehensive view of antibiotic resistance in this WWTP.

**Methods:**

18 ESBL-producing *E. coli* strains were isolated from a WWTP located in Taizhou, China on April, 2017. All strains were sequenced using Illumina HiSeq 2000 sequencer. The whole genome sequences were assembled using SPAdes software and annotated with RAST server. Sequence types (STs), plasmids, ARGs and virulence genes were predicted from the genomes using MLST, Plasmid Finder, ResFinder and Virulence Finder, respectively. Metagenomic DNA of the same sample was extracted and sequenced using Illumina Hiseq X Ten platform. Metagenomic sequences were assembled using SOAPdenovo software.

**Results:**

All 18 ESBL-producing *E. coli* strains were resistant to ampicillin, cefazolin, and ceftriaxone. Analysis of their genomes revealed that all strains carried beta-lactamase encoding genes and the most prevalent type was *bla*_*CTX–M*_. Various virulence genes and ARGs confronting resistance to other types of antimicrobial agents were also predicted. Further investigation on the metagenomics data indicated 11 ARGs with high amino acid identities to the known ARGs. Five of these ARGs, *aadA1*, *aac(6′)-lb-cr*, *flo(R)*, *sul2* and *sul1*, were also present in the genomes of the ESBL-producing *E. coli* isolated from the same sample.

**Conclusion:**

Our study revealed the resistome of a pharmaceutical WWTP by both culture-dependent and metegenomic methods. The existence of ESBL-producing *E. coli* strains, indicating that pharmaceutical WWTP can play a significant role in the emergence of ARB. The occurrence of ARGs annotated from the metagenomic data suggests that pharmaceutical WWTP can play a significant role in the emergence of ARGs. Our findings highlight the need for strengthening the active surveillance of ARB and ARGs from pharmaceutical industry.

## Introduction

The rapid emergence and global spread of antimicrobial resistant bacteria (ARB) worldwide has long been recognized as a threat to human health, and one of the most studied bacterial groups is extended-spectrum beta-lactamase (ESBL)-producing Enterobacteriaceae (Bush and Fisher, 2011). An epidemiological study has reported that ESBL-producing Enterobacteriaceae, especially *Escherichia coli*, is an important cause of community-acquired infection (Stadler et al., 2018). ESBL enzymes produced by resistant strains are capable of inactivating beta-lactam drugs (e.g., third-generation cephalosporins) by hydrolyzing their beta-lactam rings (Bush and Bradford, 2016). Some of these ESBL-producing *E. coli* may also carry virulence genes. The products of these virulence genes enhance their pathogenicity. For instance, virulence genes *eilA*, *air*, and *lphA* encode for proteins that are related to adherence of bacteria (Doughty et al., 2002; Sheikh et al., 2006). Virulence genes *senB* and *astA* encode for enterotoxins (Dubreuil et al., 2016). Some virulence genes, such as *gad*, are involved in the survival of bacteria in harsh conditions such as the extremely acidic environments in stomach (Capitani et al., 2003). Thus, the infections caused by ESBL-producing *E. coli* strains which also harbor virulence genes are always associated with increased mortality and cost burden (Schwaber et al., 2006). In addition, ESBL genes are often encoded on plasmids that can be readily exchanged between species (Carattoli, 2013). Moreover, ESBL genes have been found not only in *E. coli* strains from clinical origin, but also from environmental origin (Runcharoen et al., 2017; Zheng et al., 2017a).

Wastewater treatment plants (WWTPs) have been regarded as vast reservoirs and environmental suppliers of ARB and antimicrobial resistance genes (ARGs) (Guo et al., 2017; Jałowiecki et al., 2018). Majority of the research has primarily focused on municipal WWTP receiving hospital and household wastewater (Yu et al., 2016; Szekeres et al., 2017; Galler et al., 2018). In recent years, the issue of pharmaceuticals in the environment has been gaining more attention worldwide and there has been growing interest in the WWTPs treating effluents from pharmaceutical industries with antimicrobial agents production. For example, several studies revealed the environmental impacts and the resistome of antimicrobial agents contaminated effluents from pharmaceutical industries in India and Croatia (Fick et al., 2009; Rutgersson et al., 2014; Bielen et al., 2017; Razavi et al., 2017; González-Plaza et al., 2018). A recent study in Nigeria has applied a culture-depend method to isolate ARB in wastewater from pharmaceutical facilities (Obayiuwana et al., 2018). In China, studies have revealed the existence of ARGs in WWTPs that treated wastewater from antimicrobial agents producing factories (Li et al., 2010; Zhai et al., 2016; Tang et al., 2017). It is suggested that environmental pollution from pharmaceutical manufacturing can promote the spread of antimicrobial resistance, the induction of direct biostatic or bio-toxic effects to diverse organisms, and the alteration of species distribution (Bielen et al., 2017). Thus, there is a clear need for more research in this area.

The aim of the present study was to investigate the resistome in a WWTP of pharmaceutical industry in China. We used whole genome sequencing (WGS) to provide insight into genomic determinants of the ESBL-producing *E. coli* strains isolated from this WWTP and reveal their sequence types (STs), ARGs, virulence genes and plasmids. In addition, we applied a metagenomic approach to determine the microbial diversity and the occurrence of ARGs in this WWTP. The importance and originality of this study are derived from the identification of ARB and ARG in pharmaceutical WWTP using both WGS and metagenomic sequencing. Ultimately, it is hoped that this research will contribute to a deeper understanding of the ARB and ARGs released from pharmaceutical WWTP.

## Materials and Methods

### Sample Collection

Sludge samples were collected from a WWTP located in Taizhou, China. This WWTP employed a conventional activated sludge process to treat wastewater from a beta-lactam antibiotics manufacturing company. A number of antimicrobial agents were produced by this company, including cefaclor, ceftizoxime, ceftibuten, cefuroxime sodium, cefprozil, cefdinir, and cefixime. There was an ongoing production at the time of sampling. Three independent samples (500 g for each sample) of aerobic active sludge were collected via the inspection opening of WWTP aeration tank in April 2017. All samples stored in sterilized tubes were transported to the laboratory on ice within 24 h.

### Isolation and Antimicrobial Susceptibility Testing of ESBL-Producing *E. coli*

For pre-enrichment step, the samples were cultured overnight in Mueller-Hinton broth without antimicrobial agents. Subsequently, 500 μl enriched sample were spread on chromID^®^ ESBL (bioMérieux, France) plates (the selective chromogenic medium for ESBL-producing enterobacteria screening). The plates were cultivated at 37°C under aerobic conditions. *E. coli* colonies were picked up from each plate according to the manufacturer’s instructions (pink/burgundy) after 48 h. All these strains were re-cultivated on chromID^®^ ESBL plates for confirmation. The identification of isolated bacteria was carried out using a Microflex MALDI-TOF mass spectrometer (Bruker Daltonics, Germany) as described previously (Zheng et al., 2017b). Antimicrobial susceptibility testing was determined on all strains by VITEK 2 system employing panel AST-GN-16 (bioMérieux, France). The results were then interpreted according to the standards of the Clinical and Laboratory Standards Institute (Clsi., 2017). *E. coli* ATCC 25922 was used as control.

### Whole Genome Sequencing and Bioinformatic Analysis

Genomic DNA of the strains were extracted using QIAamp DNA Mini Kit (Qiagen, Germany) and were sent to Novogene Bioinformatics Technology, Co., Ltd. (Beijing, China) immediately on dry ice for WGS. Sequencing was carried out using the Illumina Hiseq 2000 sequencer (Illumina, United States) with a high-throughput 2 × 100 bp pair end sequencing strategy. Reads were filtered as described previously (Zheng et al., 2017b) and the resulting clean reads were assembled using SPAdes software (Bankevich et al., 2012). The draft genomes were annotated by RAST server (Aziz et al., 2008), while the 16S rRNA of strains were identified using EzBioCloud (Yoon et al., 2017). STs of the strains were predicted using MLST 2.0 (Larsen et al., 2012). Plasmids, ARGs and virulence genes were predicted from the genomes using PlasmidFinder 2.0 (Carattoli et al., 2014), ResFinder 3.1 (Zankari et al., 2012) and Virulence Finder 2.0 (Joensen et al., 2014) with nucleotide identity threshold of 90% and minimum length of 60%, respectively. Average nucleotide identity (ANI) between strains was calculated using their genomic sequences by Orthologous Average Nucleotide Identity Tool (OAT) with OrthoANI algorithm (Lee et al., 2016).

### Metagenomic DNA Extraction

Metagenomic DNA was extracted from the samples upon arrival at the laboratory. DNA extraction was performed using FastDNA^®^ Spin Kit for Soil (MP Biomedicals, Santa Ana, CA, United States) according to the manufacturer’s instructions. The quality and integrity of DNA samples were checked by 1% agarose gel electrophoresis. Meanwhile, DNA yield and purity were measured using NanoDrop^TM^ 2000 spectrophotometer (Thermo Fisher Scientific, Waltham, MA, United States) and Qubit^®^ 2.0 fluorometer (Thermo Fisher Scientific, Waltham, MA, United States). After extraction, three DNA samples were mixed into one sample prior to metagenomic sequencing.

### Metagenomic Sequencing and Bioinformatic Analysis

Metagenomic DNA was transported to Zhejiang Tianke High-Tech Development, Co., Ltd. (Hangzhou, China) on dry ice immediately after exaction. High-throughput sequencing was conducted using Illumina Hiseq Xten platform with PE150 (pair-end sequencing, 150 bp reads) sequencing mode. Raw reads generated from the sequencing were filtered by removing the reads with more than 40 bp low quality nucleotides, quality value lower than 38, more than 10 ambiguous nucleotides, and more than 15 bp overlapped with adapter. After filtering, the remaining clean reads were assembled using SOAPdenovo2 software (Luo et al., 2012). Different K-mer sizes (49, 55, and 59) were chosen for the assembly with the parameters of -d 1, -M 3, -u and -F, while the highest scaffold N50 value was selected for further analysis. The obtained scaffolds were then broken down at position N, resulting in the formation of continuous sequences within scaffolds called Scaftigs. Then, all the clean reads were aligned to Scaftigs using SOAPaligner software (Gu et al., 2013). Only Scaftigs longer than 500 bp were retained for further analysis. Open reading frames (ORFs) were predicted from the assembled Scaftigs using MetaGeneMark with default parameters (Zhu et al., 2010). CD-HIT was used to obtain a non-reduntant gene catalog (Fu et al., 2012). The retained clean reads were aligned to the gene catalog by using SOAPaligner, in order to calculate the number of matching reads (Gu et al., 2013). Unigenes were then obtained by removing the gene catalogs with less than three mapped reads. These unigenes were aligned against NCBI-NR database by DIAMOND software with the parameters of blastp, e-value ≤ 1e-5 (Buchfink et al., 2015) and further annotated with LCA algorithm using MEGAN (Huson et al., 2016). Further, ARGs were predicted by ResFinder 3.1 with 90% identity cutoff and 60% minimum length (Zankari et al., 2012).

## Results

### Antimicrobial Resistance Profiles of *E. coli* Strains

A total of 18 pink colonies were picked up after spreading the samples on chromID^®^ ESBL agar plates. By MALDI-TOF MS and 16S rRNA sequence analysis, all strains were identified as *E. coli*. As shown in Table 1, all strains were resistant to ampicillin, cefazolin and ceftriaxone, but not tigecycline.

**TABLE 1:** MIC values of all 18 *E. coli* strains.

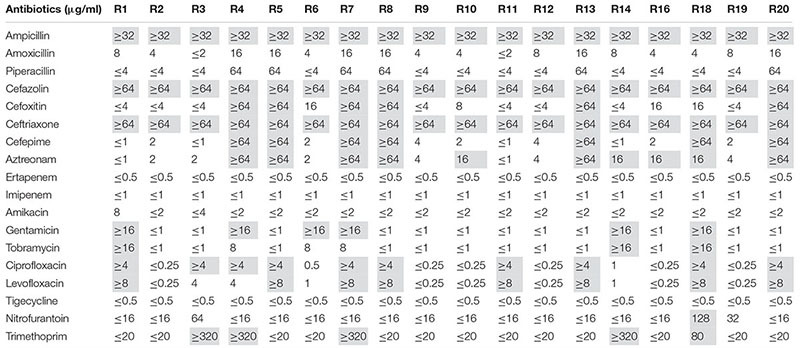

*The MIC values labeled with gray background indicate the resistance to the antimicrobial agents.*

### Genomic Features of *E. coli* Strains

Draft genome sequences of 18 *E. coli* strains were obtained through WGS and assembling. The sequences had been deposited in DDBJ/ENA/GenBank under bioproject (PRJNA433283) with an individual accession number. Genome sizes ranged from 4.67 to 5.33 Mb, with an estimated GC content between 50.3 and 50.9%. A total of 4,576 to 5,240 coding sequences, 92 to 118 RNAs were annotated via RAST server. Sequence typing was conducted with the whole genome sequences of the strains, generating eight different STs (Table 2). The strains that classified to ST405 and ST155 were accounted for 55.56% (*n* = 10). The STs represented by two strains were ST1193 (*n* = 2) and ST167 (*n* = 2). The remaining strains (*n* = 4) were singletons and represented their own ST.

**TABLE 2:** ARGs and virulence genes identified in the genomes of *E. coli* strains.

**Strain**	**STs**	**Virulence genes**	**Acquired antimicrobial resistance genes of different antimicrobial classes**								
			**Beta-lactam**	**Aminoglycoside**	**Fluoroquinolone**	**Fosfomycin**	**Macrolide, lincosamide and streptogramin B (MLS)**	**Phenicol**	**Sulfonamide**	**Tetracycline**	**Trimethoprim**
R1	ST1193	*gad senB vat iha sat*	*bla_*CTX–M–*__14_ bla_*TEM–*__1__*B*_*	*aph(3″)-lb aac(3)-lld aph(6)-ld aacA4*	*aac(6′)-lb-cr*			*cmlA1*	*sul2*	*tet(A)*	
R2	ST155	*gad lpfA*	*bla_*CTX–M–*__14_*								
R3	ST450	*gad iha senB*	*bla_*CTX–M–*__27_*	*aph(3″)-lb aadA5 aph(6)-ld*			*mph(A)*		*sul1 sul2*	*tet(A)*	*dfrA17*
R4	ST167	*gad*	*bla_*CTX–M–*__15_ bla_*CTX–M–*__27_*	*aadA5 aac(3)-lla*			*mph(A) erm(B)*		*sul1*	*tet(B)*	*dfrA17*
R5	ST405	*gad eilA air*	*bla_*CTX–M–*__15_ bla_*CTX–M–*__15_*	*aph(3″)-lb aph(6)-ld*				*floR*	*sul2*	*tet(A)*	
R6	ST38	*gad astA iss eilA*	*bla_*CTX–M–*__14_*	*aadA5 aac(3)-lld*			*mph(A)*		*sul1*		*dfrA17*
R7	ST167	*gad*	*bla_*CTX–M–*__15_ bla_*CTX–M–*__27_*	*aadA5 aac(3)-lla*			*mph(A) erm(B)*		*sul1*	*tet(B)*	*dfrA17*
R8	ST405	*eilA air*	*bla_*CTX–M–*__15_*	*aph(3″)-lb aph(6)-ld*				*floR*	*sul2*	*tet(A)*	
R9	ST155	*gad lpfA*	*bla_*CTX–M–*__14_*								
R10	ST405	*gad eilA air*	*bla_*CTX–M–*__15_ bla_*CTX–M–*__15_*	*aph(3″)-lb aph(6)-ld*				*floR*	*sul2*	*tet(A)*	
R11	ST1193	*gad vat sat iha*	*bla_*CTX–M–*__14_*								
R12	ST155	*gad lpfA*	*bla_*CTX–M–*__14_*								
R13	ST405	*gad eilA air*	*bla_*CTX–M–*__15_ bla_*CTX–M–*__15_*	*aph(3″)-lb aph(6)-ld*				*floR*	*sul2*	*tet(A)*	
R14	ST88	*gad iroN mchF ireA iss celb tsh*	*bla_*CTX–M–*__14_*	*aph(4)-la aac(3)-lVa aph(3′)-la aadA2 aadA1*		*fosA3*		*floR cmlA1*	*sul2 sul3*	*tet(B)*	*dfrA12*
R16	ST1722	*eilA air astA lpfA iss*	*bla_*CTX–M–*__27_*								
R18	ST155	*gad lpfA astA*	*bla_*CTX–M–*__14_ bla_*CTX–M–*__55_ bla_*TEM–*__1__*B*_*	*aph(3″)-lb aph(6)-ld aph(3′)-lla aph(3′)-la aadA1 aph(4)-la aac(3)-lVa, aadA2*	*oqxA oqxB*	*fosA3*	*mph (A) erm (42)*	*floR cmlA1*	*sul1 sul2 sul3*	*tet(A) tet(M)*	*dfrA17*
R19	ST155	*gad lpfA*	*bla_*CTX–M–*__14_*								
R20	ST405	*gad eilA air*	*bla_*CTX–M–*__15_ bla_*CTX–M–*__15_*	*aph(3″)-lb aph(6)-ld*				*floR*	*sul2*	*tet(A)*	

### ARGs in *E. coli* Strains

**FIGURE 1:**
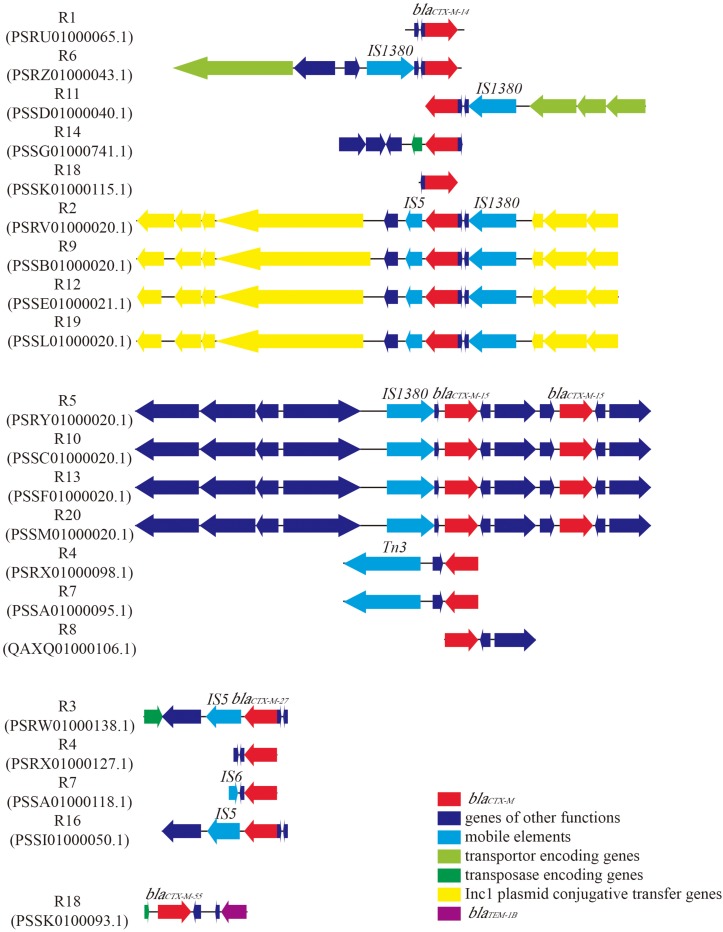
Genetic environment of *bla*_*CTX–M*_ in 18 *Escherichia coli* strains. GenBank accession numbers of the contigs which contain *bla*_*CTX–M*_ were given below the strain names. ORFs were represented by arrows. The color of the arrow indicated the function of the gene. Subtypes of *bla*_*CTX–M*_ and insertion sequence (IS) were labeled above the arrows.

In order to detect the genes corresponding to the ESBL phenotype, we screened the genome sequences of the 18 *E. coli* strains for the presence of ARGs. The results indicated that all *E. coli* strains harbored *bla*_*CTX–M*_ gene (Table 2). Further analysis of the genetic environment of *bla*_*CTX–M*_ gene revealed that strains with same STs carried the same *bla*_*CTX–M*_ subtypes. For example, Strains R2, R9, R12 and R19, designated as ST155, harbored *bla_*CTX–M–*__14_* gene, while strains R5, R10, R13 and R20, which were classified as ST405, carried two *bla_*CTX–M*__–__15_* genes and shared the same genetic environments (Figure 1). With such high similarities in gene arrangements among some strains, we wondered whether these strains were copy isolates identified in the samples. Therefore, we calculated the ANI values between the 18 isolates. The result showed that some strains shared 100% ANI, which might infer that they were actually copy isolates (Supplementary Figure S1). The genes encoding for mobile elements, transposases and plasmid conjugative transfer proteins were located near *bla*_*CTX–M*_ gene in majority of *E. coli* strains (Figure 1 and Supplementary Table S1). Besides beta-lactamase genes, most strains also carried genes conferring resistance to many other classes of antimicrobial agents. For instance, 12 strains harbored aminoglycoside resistance genes with *aph(3″)-lb* and *aph(6)-ld* as the most abundant types; sulfonamide resistance genes (e.g., *sul1, sul2*, or *sul3*) were found in 12 strains. Eleven strains carried genes encoding for tetracycline resistance, including *tet(A)*, *tet(B)*, *tet(D)*, and *tet(M)*. Moreover, resistance genes conferring resistance to phenicols, trimethoprims, macrolides, lincosamides and streptogramin B (MLS), fluoroquinolones, and fosfomycins were detected in some of the strains.

### Virulence Genes in *E. coli* Strains

Virulence genes were detected in all *E. coli* strains and the numbers of virulence genes were different among the strains with different STs. One ST88 strain, R14, that encoded seven virulence genes (*gad*, *iroN*, *mchF*, *ireA*, *iss*, *celb*, and *tsh*), harbored more virulence genes than other STs strains. While the two ST167 strains, R4 and R7, only harbored *gad* gene. Of all the virulence genes, the glutamate decarboxylase encoding gene *gad* was the most common virulence gene presented in the 16 out of 18 strains (88.89%) (Table 2). The second predominant type was *eilA* (encoding for Salmonella HilA homolog), followed by *air* (encoding for Enteroaggregative immunoglobulin repeat protein) and *lpfA* (encoding for long polar fimbriae). Two enterotoxin genes, namely, *astA* and *senB*, were found in some *E. coli* strains.

### Plasmids in *E. coli* Strains

17 out of 18 strains were predicted to harbor plasmids by PlasmidFinder (Supplementary Table S2). The Col156 plasmid replicon type was observed to be the most prevalent type among these strains (*n* = 13). The five *E. coli* ST405 strains (R5, R8, R10, R13, and R20) carry one Col(MP18) plasmid replicon type and one IncY plasmid incompatibility groups, with strain R5 and R13 additionally harboring one Col(156) replicon type. The four *E. coli* ST155 strains (R2, R9, R12, and R19) were identified to carry the same four plasmids: Col(MP18), Col(156), IncB/O/K/Z and IncY. However, another ST155 strain, R18, was identified to carry eight plasmids [Col(MG828), IncFII(pHN78A), IncHI2, IncHI2A, IncI1, InclQ1, IncX1, and P0111].

### ARGs in WWTP

By metagenomics sequencing, a total of 109,523,076 clean reads were generated and were then deposited in NCBI Sequence Read Archive with accession number of SRP132643. 11 ARGs with high identities were annotated using the assembled sequence generated by metagenomic sequencing. These ARGs are involved in the resistance to various types of antimicrobial agents including aminoglycosides, fluoroquinolones, macrolides, phenicols, and sulfonamides (Supplementary Table S3). Five ARGs identified in metagenomic data were also carried by the *E. coli* strains isolated from the same WWTP (Table 2).

## Discussion

In recent years, a growing number of studies have shown that WWTPs may function as major reservoirs of ARB and ARGs (Conte et al., 2017; Guo et al., 2017; Szekeres et al., 2017). Although most studies have reported ARB and ARGs from municipal and hospital WWTP, some researchers focused on the pharmaceutical industry WWTPs for the wastewater discharged from pharmaceutical industry may contain high concentrations of hazardous substances, including antimicrobial agents. High abundance of resistant strains has been observed in pharmaceutical WWTPs. In particular, 63.6 ± 6.4% of the bacterial strains isolated from penicillin production wastewater (Li et al., 2009) and 86% of the bacterial strains from a WWTP of multiple bulk drug manufacturers (Marathe et al., 2013) were resistant to many antimicrobial agents. Notwithstanding this, most of the bacterial strains isolated from these WWTPs were non-pathogenic. For instance, only two strains were identified as *E. coli* among the 189 strains isolated from a WWTP treating oxytetracycline, avermectin, and ivermectin production wastewater (Li et al., 2010). Therefore, in our study, we used selective plates for the isolation of ESBL-producing *E. coli* from the pharmaceutical WWTP. In total, we recovered 18 ESBL-producing *E. coli* strains. The results of antimicrobial susceptibility testing demonstrated that all strains displayed resistance to beta-lactams such as ampicillin, cefazolin, and ceftriaxone (Table 1).

In order to investigate the ARGs carried by these strains, WGS technology was applied to retrieve the draft genome sequences of all the 18 strains. Not surprisingly, all of the strains were found to carry at least one beta-lactamase gene, and the most prevalent ARGs type was *bla*_*CTX–M*_ (Table 2). Previous studies have demonstrated that *bla*_*CTX–M*_ is the most common ARGs type in ESBL-producing *E. coli* strains of human (Campos et al., 2018) and animal origin (Wu et al., 2018). The molecular homology analysis of *bla*_*CTX–M*_ positive *E. coli* strains further suggested a transmission of pathogens across water, food animals and human (Hu et al., 2013). Our finding in accordance with other studies and revealed that *bla*_*CTX–M*_ was the most dominant ARGs type in WWTPs of industrial origin. The results of sequence alignments indicated *bla*_*CTX–M*_-containing contigs share a high degree of similarity among the strains with the same STs (Figure 1 and Table 2). Further investigation of genetic environment revealed that the genes encoding for mobile elements, transporters, transposase and Incl1 plasmid conjugative transfer proteins located near the *bla*_*CTX–M*_ in most of the strains, suggesting the transfer potential of *bla*_*CTX–M*_ (Figure 1). In addition, plasmid prediction revealed that *bla_*CTX–M–*__14_* genes and IncB/O/K/Z plasmids were located in the same contigs of strains R2, R9, R12, and R19. Likewise, *bla_*TEM–*__1__*B*_* gene and plasmid IncFIA were predicted in the same contig of strain R1. Further indicating the transfer potential of these ARGs via plasmids.

Numerous virulence genes were detected in the genomes of the 18 ESBL-producing *E. coli* strains. For instance, all ST405 strains (R5, R8, R10, R13, and R20) harbored *eilA* and *air* genes which encoded for Salmonella HilA homolog and Enteroaggregative immunoglobulin repeat protein, respectively. The long polar fimbriae encoding gene *lpfA* was detected in all ST155 strains (R2, R9, R12, R18, and R19). These genes were demonstrated to play a role in the adherence of the bacteria (Doughty et al., 2002; Sheikh et al., 2006). Strain R14 which belonged to ST88, harbored the highest number of virulence genes among all the strains. The possession of a high number of virulence traits in ST88 stains was also reported in previous studies (Oteo et al., 2014). Among these virulence genes, *gad* was the most prevalent type (Table 2). The *gad* gene enables the bacteria to survive in harsh acidic conditions in gastric environment by helping them to maintain a near-neutral intracellular pH (Capitani et al., 2003). Genes encoded for enterotoxins were also discovered. Strain R1 and R3 carried *senB* gene which encoded for plasmid-encoded enterotoxin. While strain R6, R16, and R18 harbored the heat-stable enterotoxin 1 encoding gene *astA*. These enterotoxins are responsible for fluid secretion and may associated with diarrhea (Dubreuil et al., 2016). With all these virulence genes, the *E. coli* strains isolated from the pharmaceutical WWTP may pose a potential threat to human health.

In addition to metagenomic sequencing, numerous studies have employed quantitative polymerase chain reaction (qPCR) for the detection of ARGs and their relative abundance (Szekeres et al., 2017; Zheng et al., 2017c). However, due to the limited availability of qPCR primers, metagenomic sequencing may be more appropriate for the screening of both known and novel ARGs (Schmieder and Edwards, 2012; Berglund et al., 2017; Marathe et al., 2018). Nevertheless, a combination of WGS and metagenomic sequencing can be an effective strategy to unravel the resistome in WWTP. To further reveal ARGs in this WWTP, a high-throughput metagenomic sequencing technology was implemented. The occurrence of 11 ARGs was analyzed from the metegenomic sequence with high amino acid identities (Supplementary Table S3). Five of these ARGs, *aadA1*, *aac(6′)-lb-cr*, *flo(R)*, *sul2* and *sul1*, were also present in the genomes of the *E. coli* isolated from the same WWTP. However, we failed to find any beta-lactam resistance genes in the metagenomic data, while these beta-lactam resistance genes were carried by all the 18 *E. coli* isolated from the same sample. That may due to the low abundance of these beta-lactam resistance genes in the metagenomics data. By taxonomy annotation, only three out of 105,892 unigenes were annotated as *E. coli*, indicating that *E. coli* was of very low abundance in the WWTP. This low abundance of *E. coli* in WWTP was also observed in previous study (Li et al., 2010). Only those *E. coli* that exhibit antimicrobial resistance can survive in this WWTP which treats the antimicrobial agents producing pharmaceutical wastewater. The present work reported the isolation and analysis of ESBL-producing *E. coli* in a pharmaceutical WWTP and presented their antimicrobial phenotypes as well as genotypes. However, the background of *E. coli* in this geographic area is still unknown. In order to draw a whole picture of the prevalence of ESBL-producing *E. coli* and to evaluate the potential risk of their transmission in this area, further study needs to be done to elucidate the diversity of *E. coli* in this area.

## Conclusion

In present study, we applied both WGS and metagenomics sequencing technologies to uncover the antimicrobial resistance profiles of ESBL-producing *E. coli* strains and the antimicrobial resistome in a pharmaceutical WWTP. Our findings indicate that pharmaceutical WWTP can play a significant role in the emergence of ARB and ARGs. In order to prevent the spread of ARB and ARGs, the sewage sludge produced by this WWTP are treated through dewatering and incineration. Thus, further research is required to investigate the ARB and ARGs in the sludge dewatering streams.

## Author Contributions

XJ and BZ designed the study. XC and HX performed the experiments. WL, FT, TS, and XP contributed reagents, materials, and analysis tools. XJ and BZ analyzed the data and wrote the manuscript. All authors revised the manuscript.

## Conflict of Interest Statement

The authors declare that the research was conducted in the absence of any commercial or financial relationships that could be construed as a potential conflict of interest.
