# Supplementary figures and images for: Whole Genome Sequencing of Extended-Spectrum Beta-Lactamase (ESBL)-Producing Escherichia coli Isolated From a Wastewater Treatment Plant in China
Source: Front Microbiol. 2019 Aug 2;10:1797. doi: 10.3389/fmicb.2019.01797 (PMC6688389; doi:10.3389/fmicb.2019.01797)

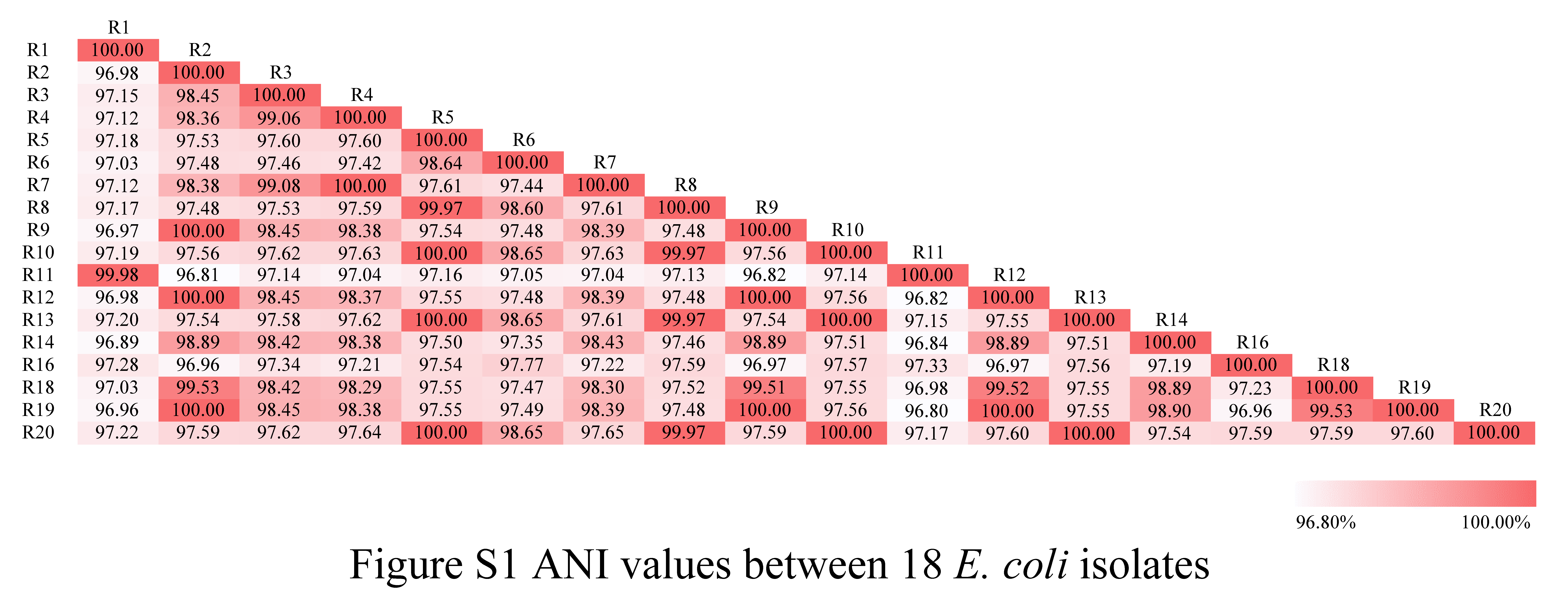

Supplement: Supplementary file 1 [file Image_1.TIFF]
