# Supplementary material for: Whole Genome Sequencing of Extended-Spectrum Beta-Lactamase (ESBL)-Producing Escherichia coli Isolated From a Wastewater Treatment Plant in China
Source: Front Microbiol. 2019 Aug 2;10:1797. doi: 10.3389/fmicb.2019.01797 (PMC6688389; doi:10.3389/fmicb.2019.01797)
Supplement: Supplementary file 3 [file Table_2.DOCX]

| Strain | Plasmids |
| --- | --- |
| R1 | IncFIA, IncFIB (AP001918),Col(BS512),Col156 |
| R2 | Col(MG828), Col156, IncB/O/K/Z,IncX4 |
| R3 | Col(MG828), Col156, IncFIB (AP001918), IncFII (29) |
| R4 | Col(BS512), Col(MG828), Col156, IncFIA, IncFIB (AP001918), IncFII, IncFII(pSE11) |
| R5 | Col(MP18), Col156, IncY |
| R6 | NOT FOUND |
| R7 | Col(BS512), Col(MG828), Col156, IncFIA, IncFIB (AP001918), IncFII, IncFII(pSE11) |
| R8 | Col(MP18), IncY |
| R9 | Col(MG828), Col156, IncB/O/K/Z, IncX4 |
| R10 | Col(MP18), IncY |
| R11 | Col(BS512), Col(MG828), Col156, ColRNAI, IncFII(pSE11), IncI1 |
| R12 | Col(MG828), Col156, IncB/O/K/Z, IncX4 |
| R13 | Col(MP18), Col156, IncY |
| R14 | Col156, Col440II, IncFIB (AP001918), IncFIC(FII), IncHI2, IncHI2A |
| R16 | Col(BS512), Col(MG828), Col156, Col8282, IncFIB (AP001918), IncFII(29), Incl1 |
| R18 | Col(MG828), IncFII(pHN78A), IncHI2, IncHI2A, IncI1, InclQ1, IncX1, P0111 |
| R19 | Col(MG828), Col156, IncB/O/K/Z, IncX4 |
| R20 | Col(MP18), IncY |

Table S2 Plasmids predicted from the 18 *E. coli* strains
