# Supplementary material for: Whole Genome Sequencing of Extended-Spectrum Beta-Lactamase (ESBL)-Producing Escherichia coli Isolated From a Wastewater Treatment Plant in China
Source: Front Microbiol. 2019 Aug 2;10:1797. doi: 10.3389/fmicb.2019.01797 (PMC6688389; doi:10.3389/fmicb.2019.01797)
Supplement: Supplementary file 4 [file Table_3.DOCX]

Table S3. Acquired antimicrobial resistance genes predicted in WWTP

| Resistance gene | Identity (%) | Resistance type |
| --- | --- | --- |
| *aad*A6 | 100.00 | Aminoglycosides |
| *aad*A1 | 100.00 | Aminoglycosides |
| *aac(6’)-lla* | 99.64 | Aminoglycosides |
| *aac(6’)-lb3* | 99.82 | Aminoglycosides |
| *ant(2”)-la* | 100.00 | Aminoglycosides |
| *aac(6’)-lb-cr* | 99.48 | Fluoroquinolones and aminoglycosides |
| *erm(F)* | 99.75 | Macrolides |
| *floR* | 94.96 | Phenicols |
| *catB4* | 100.00 | Phenicols |
| *sul2* | 100.00 | Sulphonamides |
| *sul1* | 99.88 | Sulphonamides |
